# New Jersey

**Published:** 1896-04

**Authors:** 


### NEW JERSEY.

An Act to establish a State Board of Animal Industry and to provide for the regulation, control, and suppression of contagious and infectious diseases of domestic animals, introduced by Mr. Parry, of the Senate, and referred to the Committee on Agriculture and Agricultural College:

Section 1. Composition of Board: President and Secretary of the State Board of Agriculture and five other members, the latter to be appointed by the President.

Sec. 2 prescribes the duties of the Board to investigate as to the existence of contagious, dangerous, or infectious diseases, provides for their suppression, and empowers them to establish quarantine measures when required; the disinfection of premises and localities.

Sec. 3 deals with the condemnation of animals and the method of appraisement; three-fourths of the appraised value will be paid by the State, based upon the actual value and condition at the times of appraisement; no appraisement to exceed eighty dollars for a registered animal; forty dollars for all others.

SEC. 4 confers upon the Board the right to enter any place or premises in the performance of their duties.

SEC. 5 provides for the employment of a Secretary and other assistants and agents and the purchase of supplies, to administer oaths or affirmation to appraisers, and the power to direct such examinations into the condition of the live stock as they may deem necessary.

SEC. 6 provides for a record being kept of all expenses, and its being printed annually in the report of the State Board of Agriculture.

SEC. 7 carries with it an annual appropriation of six thousand five hundred dollars for expenses and the payment for slaughtered stock; no other compensation to be allowed the Board other than their expenses.

SECS. 8, 9, and 10 provide for audit of expenses and payment by State Treasurer, rescinding all acts or parts of acts inconsistent with this act, and that it shall take effect immediately.

The bill introduced in the House to again reopen and extend the time of registration for non-graduates was so strongly opposed by some of the leading veterinarians of the State that it failed to receive favorable consideration at the hands of the committee, and therefore did not reach the calendar.

The bill to afford cities the power to create city veterinarians by ordinance of city council was favorably reported back to the House and placed on the calendar, and it is hoped that the session will not close without its successful passage in both branches. This bill was defeated in the closing hours of the session after a heroic battle to save it.

The bill creating a State Board of Animal Industry has met with much opposition from the present Tuberculosis Commission, whose powers and authority were to be relegated to the new Bureau. Inasmuch as it failed to provide for a single representative of the veterinary profession on the same, whose qualifications are such that no properly equipped board can longer be complete without such a representative, there will be little regret if this bill fails to become a law at the present session.

The bill introduced in the Assembly looking to the creation, by ordinance of councils, of city veterinarians in the several cities in New Jersey, was defeated in the final struggle at the close of the session at Trenton. The bill looking to a reopening of the registration-books to non-graduates failed to emerge from the committee to which it was referred, and, therefore, did not become a law. The third act, taking the work of management of tuberculosis and other contagious and infectious diseases among domestic animals from the Tuberculosis Commission and placing it in the hands of a State Bureau of Animal Industry to be composed wholly of the laity, failed also in passage.

